# Multiomics and Artificial Intelligence for Personalized Nutritional Management of Diabetes in Patients Undergoing Peritoneal Dialysis

**DOI:** 10.1016/j.advnut.2025.100378

**Published:** 2025-01-20

**Authors:** Sara Mahdavi, Nicole M Anthony, Tabo Sikaneta, Paul Y Tam

**Affiliations:** 1 Department of Nutrition, Harvard T. H. Chan School of Public Health, Boston, MA, United States; 2 Department of Nutritional Sciences, University of Toronto, 6 Queen’s Park Cres, Toronto, Ontario, Canada; 3 Department of Nephrology, the Scarborough Health Network, Toronto, Ontario, Canada; 4 Kidney Life Sciences Institute, Toronto, Ontario, Canada

**Keywords:** precision nutrition, kidney disease, omics, diabetes, peritoneal dialysis, artificial intelligence

## Abstract

Managing diabetes in patients on peritoneal dialysis (PD) is challenging due to the combined effects of dietary glucose, glucose from dialysate, and other medical complications. Advances in technology that enable continuous biological data collection are transforming traditional management approaches. This review explores how multiomics technologies and artificial intelligence (AI) are enhancing glucose management in this patient population. Continuous glucose monitoring (CGM) offers significant advantages over traditional markers, such as hemoglobin A1c (HbA1c). Unlike HbA1c, which reflects an mean glucose level, CGM provides real-time, dynamic glucose data that allow clinicians to make timely adjustments, leading to better glycemic control and outcomes. Multiomics approaches are valuable for understanding genetic factors that influence susceptibility to diabetic complications, particularly those related to advanced glycation end products (AGEs). Identifying genetic polymorphisms that modify a patient's response to AGEs allows for personalized treatments, potentially reducing the severity of diabetes-related pathologies. Metabolomic analyses of PD effluent are also promising, as they help identify early biomarkers of metabolic dysregulation. Early detection can lead to timely interventions and more tailored treatment strategies, improving long-term patient care. AI integration is revolutionizing diabetes management for PD patients by processing vast datasets from CGM, genetic, metabolic, and microbiome profiles. AI can identify patterns and predict outcomes that may be difficult for humans to detect, enabling highly personalized recommendations for diet, medication, and dialysis management. Furthermore, AI can assist clinicians by automating data interpretation, improving treatment plans, and enhancing patient education. Despite the promise of these technologies, there are limitations. CGM, multiomics, and AI require significant investment in infrastructure, training, and validation studies. Additionally, integrating these approaches into clinical practice presents logistical and financial challenges. Nevertheless, personalized, data-driven strategies offer great potential for improving outcomes in diabetes management for PD patients.

**Statements of significance**

The review highlights novel approaches that combine multiomics technologies with artificial intelligence to personalize the nutritional management of diabetes in patients undergoing peritoneal dialysis (PD). Specifically, we highlight how continuous glucose monitoring, alongside genomic, metabolomic, and microbiome data, can enable highly individualized treatment plans, addressing unique glycemic control challenges in this patient population—a significant advancement beyond traditional hemoglobin A1c monitoring and standard PD protocols.

## Introduction

**FIGURE 1:**
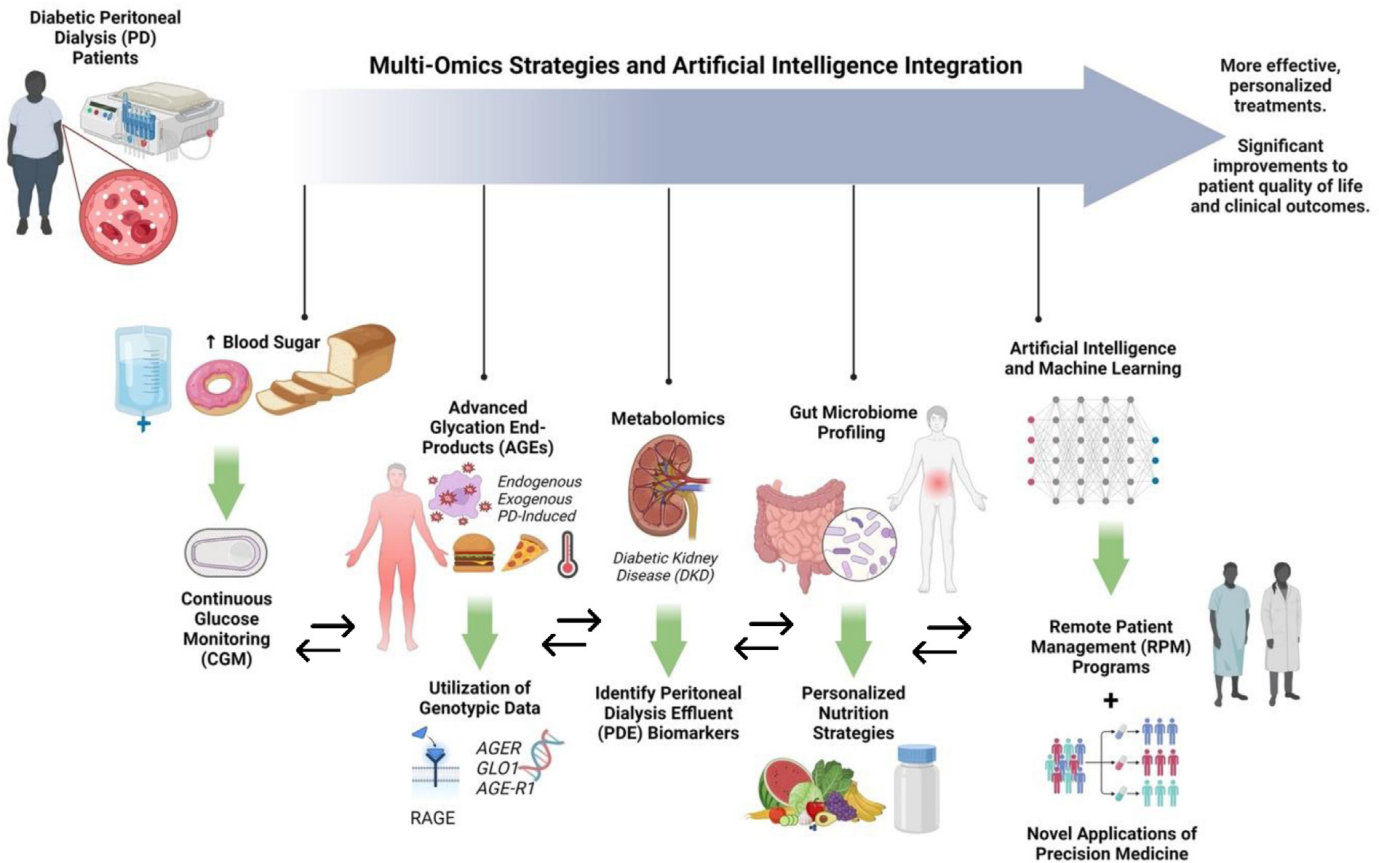
Multiomics and AI integration in diabetic PD management. The integration of multiomics strategies and AI in the treatment of diabetes in patients receiving PD treatment aims to enhance personalized treatments and significantly improve patient quality of life and clinical outcomes. The multiomics strategies include CGM for effective glucose management, utilization of genetic testing to identify individuals with a higher risk of susceptibility to tissue damage from AGEs in diabetic complications, metabolomics for identifying PDE biomarkers in DKD, and gut microbiome profiling to develop personalized nutrition strategies. Additionally, AI and ML are employed to implement RPM programs, optimizing healthcare outcomes through a comprehensive and individualized approach for managing diabetes in patients undergoing PD. Abbreviations: AGEs, advanced glycation end products; AI, artificial intelligence; CGM, continuous glucose monitoring; DKD, diabetic kidney disease; PD, peritoneal dialysis; PDE, peritoneal dialysis effluent; ML, machine learning; RPM, remote patient management.

Approximately 10% of the adult population worldwide has chronic kidney disease (CKD), and over 2,000,000 people with end-stage renal disease (ESRD) worldwide receive lifesaving dialysis, with >80% of these patients living in affluent countries [1]. Among the dialysis options, peritoneal dialysis (PD) is lower-cost [2] and home-based, which requires the patient or caregiver to manage many aspects of the treatment at home on a regular and cyclical daily schedule. Patients who receive PD compared with hemodialysis (HD) tend to be younger, of non-Hispanic White ethnicity, married, more physically independent, have higher education, live with others, and have fewer pre-existing morbidities [3]. In North America, PD is used by 9% of patients with ESRD in the United States, 25% in Canada, and 58% in Mexico [1]. Diabetes is the major cause of ESRD [4] and accounts for 45% of incident cases in North America [5]. Patients with diabetes and ESRD have significantly higher mortality and morbidity compared with those with either condition alone. Patients with diabetes receiving PD treatment face unique challenges in self-managing their diseases with a higher lifestyle modification, medication, and treatment burden compared with in-center HD patients who can rely more on specialized healthcare professionals to help with their life-sustaining dialysis treatments [5]. For example, in addition to having to manage hyperglycemia from dietary carbohydrates, these patients also must contend with significant carbohydrate loads from glucose-based PD solutions [6]. Glycemia management in ESRD is further compounded by the limitations of self-monitoring blood glucose and hemoglobin A1c (HbA1c) levels [4] that do not accurately support real-time glucose control in this population. Recent advances in multiomics technologies and continuous glucose monitoring (CGM) offer new insights and opportunities for personalized dietary and glycemia management strategies to improve outcomes and quality of life for patients with diabetes undergoing PD (Figure 1).

## Nutritional Needs and Dietary Challenges

Diet plays a crucial role in prevention, treatment, and the overall management of CKD and diabetes and is particularly challenging in diabetic patients receiving PD. As CKD progresses to ESRD, disruptions in protein and energy balance occur [7], along with altered acid–base regulation [8] and hormonal dysfunction [9] leading to complications such as bone mineral disease [10] and anemia [11]. Nephrologists and renal dietitians work closely with patients to manage these issues through a combination of nutrition and medications. However, continued declines in kidney function lead to nitrogenous waste accumulation from protein catabolism that can impair taste and appetite, mediated by genetic differences in taste perception [12], whereas uremia affects gut microbiota [13] and disrupts nutrient absorption. Muscle wasting often develops as CKD progresses to ESRD [14], with an accelerated aging effect [15] that resembles the decline in physical function and independence in elderly individuals [16]. Protein-energy wasting is common and necessitates tailored dietary interventions and nutritional therapy that not only helps manage complications, such as electrolyte imbalances and bone disorders, but may also delay the need for dialysis [17]. After dialysis initiation, dietary management remains essential for many aspects of disease management in dialysis patients [18]. For example, dietary protein-energy intake [19], sodium [20,21], calcium [22], magnesium [23,24], selenium [25], phosphate [26], potassium [27], fluids [28], water-soluble vitamins [29], and vitamin D [30] each require special consideration and management.

The glucose used in PD solutions, which acts as an osmotic agent for fluid removal, presents an additional challenge for this patient population. This glucose is absorbed directly into the bloodstream, potentially leading to continuous increases in blood glucose levels, especially during ongoing dialysis sessions. This effect is particularly significant in patients known as high transporters [31], where glucose is rapidly absorbed from the dialysate, potentially leading to severe hyperglycemia. In such cases, adjustments to glucose concentrations in dialysis solutions can be used as a management strategy. Alternatively, osmotic agents, such as icodextrin, can be used during long dwell times to minimize glucose absorption and manage hyperglycemia effectively [32]. Dietary carbohydrates impact blood glucose levels based on their glycemic index and the body’s metabolic response [6]. Foods with a high glycemic index may cause rapid spikes in blood glucose levels, whereas foods with a low glycemic index led to more gradual and controlled increases. Effective management of diabetes requires careful meal planning to synchronize carbohydrate intake with dialysis schedules and insulin therapy, ensuring stable blood glucose levels throughout the day. Additionally, dietary recommendations often focus on balancing protein intake [33] and incorporating sufficient dietary fiber to enhance gut health and reduce uremic toxin production [34,35]. Consequently, both dietary carbohydrate management and dialysis solution composition influence glucose control and require consideration when treating diabetes in patients receiving PD treatment.

CGM offers a significant advancement in managing blood glucose levels in this patient population [[36], [37], [38]], especially when traditional measures like HbA1c fall short [4] (Figure 1). HbA1c is widely recognized as a key marker for long-term glycemic control, reflecting mean blood glucose levels over the previous 2 to 3 months. However, the reliability of HbA1c diminishes in patients undergoing dialysis due to factors like altered red blood cell turnover and erythropoietin therapy, which can skew results [36,38]. Fourth-generation CGM devices measure glucose in interstitial fluid, providing real-time data on glucose fluctuations that HbA1c cannot capture [38]. This is particularly relevant among the dialysis population, where glucose levels can be unpredictably influenced by the glucose content in peritoneal dialysates. CGM can also detect episodes of hypoglycemia and hyperglycemia, allowing for precise and more immediate adjustments in therapy [37]. This can enable tailored dietary and pharmaceutical recommendations that stabilize blood glucose levels to enhance personalized care and treatment outcomes.

## Advanced Glycation End Products and Genetics

Advanced glycation end products (AGEs) are toxic compounds formed when proteins or fats combine with sugars in the bloodstream [39]. AGE accumulation interferes with normal cell functions and leads to oxidative stress, inflammation, and the progression of cardiovascular disease, diabetes, and CKD [39]. In patients with diabetes specifically, the accumulation of AGEs during PD can further contribute to the development and progression of diabetic complications. Different sources of AGEs can also adversely impact treatment outcomes in this patient population [39,40].

Endogenous AGEs are formed naturally within the body from nonenzymatic glycation reactions between sugars and proteins. These reactions are typically regulated by the body's metabolic processes. However, persistent hyperglycemia in diabetes [40] and PD treatment [41] can increase the formation and accumulation of AGEs, contributing to metabolic complications. Exogenous AGEs, derived from dietary sources, particularly high-protein and high-fat foods cooked at high temperatures, add to the AGE burden in patients with diabetes undergoing PD (Figure 1). Since renal function is compromised in PD patients, the ability to clear these AGEs is reduced and levels accumulate [41].

The receptor for advanced glycation end products (RAGE) is a cell surface receptor implicated in the pathophysiology of various chronic diseases, such as diabetes and kidney disease, due to its interaction with AGEs [42]. Research indicates that genetic factors can influence individual susceptibility to AGE toxicity [43]. These genetic differences can affect the characteristics of the peritoneal membrane, which in turn can impact glucose absorption during PD. This is important for diabetes management in patients receiving PD, as variations in the peritoneal membrane directly impact glucose absorption and solute transport. For instance, initial solute transport status, which categorizes patients based on how quickly solutes transfer from blood to dialysate during dialysis, is a critical factor in determining the appropriate dialysis prescription and can influence outcomes for patients receiving PD [43]. Interestingly, polymorphisms in the *AGER* gene encoding RAGE have been found to influence initial transport rates, with the genotype −374 TA less likely to be associated with high initial transport compared with the TT genotype [44]. Similarly, polymorphisms in genes encoding for the IL-6 and the TIE2 receptor have been found to negatively associate with high initial transport status [45]. Both IL-6 and TIE2 receptors have been found to impact the inflammatory and structural properties of the peritoneal membrane. Since initial transport status informs dialysis treatment plans and is linked to patient outcomes—higher transport rates being associated with poorer prognoses [43]—predicting a patient's transport status through genetic variations in markers associated with AGE signaling may help tailor dialysis protocols more effectively. Moreover, utilizing patient genotypic data independently or in combination with the gold standard peritoneal equilibration test presents an opportunity to further enhance treatment plans and promote beneficial health outcomes for patients receiving PD.

Additional genetic factors may influence AGE-related complications in diabetic patients undergoing PD. Glyoxalase 1 (GLO1) acts against AGE accumulation by detoxifying precursors [46], with polymorphisms in this gene conferring differences in how effectively detoxification occurs [47]. AGE receptor 1 (AGE-R1), a cell surface receptor with effects counteractive to those of RAGE [48], exhibits downregulated expression levels in diabetic nephropathy [49], leading to reduced efficiency of AGE clearance from the body. Further studies are needed to determine whether genetic variations in AGE-R1 impact its protective effect against AGEs, and whether such genetic information could guide precision nutrition recommendations in patients undergoing PD.

Given the significant impact of AGEs on the progression of diabetes and CKD in patients receiving PD, it is important to consider genetic differences that could influence individual responses to treatment (Figure 1). Polymorphisms in genes like RAGE, GLO1, and AGE-R1 provide valuable insights into interindividual susceptibility to AGE accumulation and clearance. Integrating genotyping into clinical practice promises to enhance the management of diabetic complications during PD treatment, thereby benefiting the health of patients and their quality of life.

## Metabolites and Biomarkers

Diabetic kidney disease (DKD) occurs in ∼1/3 of persons with diabetes and is more likely with prolonged hyperglycemia. It is usually diagnosed clinically, rather than based on renal biopsy, in patients who present with albuminuria and then declining renal function. However, a clinical diagnosis is often made long after significant histopathologic changes have occurred. As such, there is an urgent need for earlier diagnostic methods, including possibly metabolomics. Since diabetes is the major cause of ESRD, advances in metabolomics technologies for monitoring and predicting changes in biomarkers present in peritoneal dialysis effluent (PDE) could also prove relevant for identifying metabolic shifts that precede and characterize peritoneal membrane dysfunction (Figure 1). A systematic review of 7 studies [50] found that several PDE biomarkers linked to peritoneal membrane dysfunction (including specific novel profiles of amino acids, amines, short-chain fatty acids (SCFAs), and phospholipid species) change in concentration well before the onset of severe complications like peritoneal fibrosis and encapsulating peritoneal sclerosis (EPS) become evident. Additionally, a targeted metabolomics analysis revealed that PD fluid supplemented with the antioxidant alanyl-glutamine was shown to reduce cellular stress and improve peritoneal transport status [50,51]. Further studies investigating metabolomic signatures in PD fluid at different stages of DKD could lead to improved peritoneal permeability assessments and early, noninvasive detection of diabetic patients at risk for PD failure and EPS. Moreover, analyzing PDE profiles could lead to optimized PD fluids and personalized treatment plans, improving patient outcomes overall.

## Gut Microbiota and Dietary Fiber

Emerging research on the human gut microbiota suggests a significant connection between dietary habits, the gut microbiome, and the pathogenesis of CKD [52]. Understanding the relationship between diet and gut health alongside their impact on the effectiveness of PD treatment opens new possibilities to develop targeted, personalized nutritional interventions aimed at improving outcomes in this patient population (Figure 1). The gut microbiota, encompassing a variety of microorganisms including bacteria, viruses, archaea, and fungi, plays a pivotal role in maintaining health. The gut microbiome is dominated by phyla, such as *Firmicutes* and *Bacteroidetes*, which are bacteria essential for metabolizing dietary components and synthesizing nutrients that are critical for energy, metabolic, and immune homeostasis [53]. Dysbiosis, or the imbalance of gut microbiota, is linked to the progression of chronic diseases, such as obesity, cardiovascular disease, diabetes, and CKD. In addition to genetics and lifestyle, diet and medications can directly influence the composition of the gut microbiome [52]. For example, metformin exerts some of its blood glucose effects through its interactions with gut bacteria such as *Megamonas* and *Klebsiella pneumoniae* [54]. This highlights the capacity of using data obtained through gut microbiota analyses to more effectively inform and tailor dietary interventions to manage diabetes in patients undergoing PD.

Disturbances in gut microbiome composition, a hallmark of dysbiosis, have been implicated in the progression of CKD and significantly increase the risk of PD failure [52]. Patients with CKD exhibit decreased gut microbial diversity [55], and patients with both diabetes and ESRD show shifts in microbiota composition that favor pathogenic gut bacteria [56,57]. During PD, patients experience chronic intestinal exposure to glucose-rich dialysate, which can serve to increase bacterial species that thrive in uremic conditions [58], a hallmark of CKD. Some pathogenic species of gut bacteria can also increase uremic toxin production and absorption. However, their abundance can be reduced through supplements of prebiotics, probiotics, postbiotics, and dietary fiber [59,60]. Diets rich in fiber have been shown to promote the growth of beneficial microbes, which produce SCFAs [61]. SCFAs play a role in maintaining gut barrier integrity and exhibit systemic anti-inflammatory effects, which have the potential to slow the progression of CKD.

Emerging findings continue to challenge previous understandings of nutrition metabolism and specific nutrient requirements. These discoveries suggest that traditional models of nutrient metabolism may not fully capture the dynamic interactions between diet, genetics, and the microbiome. A recent study of 5 multiethnic cohorts, with nearly 10,000 participants demonstrated that [62] tryptophan metabolites, particularly those from the kynurenine pathway (kynurenine, kynurenate, xanthurenate, and quinolinate), are associated with increased diabetes risk. Conversely, indolepropionate, a gut microbial metabolite of tryptophan, is inversely related to diabetes. Notably, higher intakes of fiber, rather than protein or tryptophan-rich foods, supports a favorable tryptophan metabolite profile, with fiber-using gut bacteria like *Firmicutes* [63] playing a crucial role. A key finding involved the additional interaction between host genetics, such as the Lactase Control region (LC) variant affecting lactase persistence, and gut microbiota, where lactase nonpersistent individuals with higher milk intake had elevated levels of *Bifidobacterium* and indolepropionate. This crosstalk between host genes, diet, and the microbiome suggests a significant multiomics influence on diabetes development, reinforcing the need to further investigate and incorporate gut microbial data in precision nutrition approaches for conditions like diabetes in PD patients. Managing the vast volume and variety of multiomics, dietary and clinical data required for precision nutrition present significant challenges in integrating diverse data types for clinical decision-making. AI offers a solution by efficiently processing and analyzing complex datasets, enabling the identification of meaningful patterns and relationships that can guide personalized nutrition interventions for conditions like diabetes in PD patients.

To enhance dietary interventions, advanced machine learning (ML) techniques have demonstrated how AI can deepen our understanding of gut microbiota-related conditions [64,65]. Specifically, AI has proven effective in predicting hyperuricemia, a common complication in CKD, through the analysis of microbiota composition [66]. This showcases the capability of AI to identify key bacterial taxa, which influence metabolic disturbances and aid in the tailoring of personalized dietary strategies to reduce PD risks. Given the role of dysbiosis in the progression of CKD and the poor outcomes associated with PD, targeted manipulation of the gut microbiota through personalized nutritional strategies aided by AI could prove important. These strategies, based on altering individual microbiota profiles to prevent and reverse dysbiosis, might be evaluated in the management of complications and for improving outcomes in patients with diabetes receiving PD treatment.

## Integration of AI and Advanced Monitoring Technologies

In managing diabetes in PD patients, AI is increasingly proving useful, particularly in predicting complications, optimizing treatment, and improving patient outcomes. Several studies demonstrate how AI applications enhance care for both diabetes and dialysis patients [67,68]. For instance, ML models, such as random forest and support vector machines, have been utilized to predict complications like peritonitis in PD patients and cardiovascular events in HD patients. These models leverage patient-specific data—such as blood pressure and heart rate variability—to predict the onset of complications early, allowing for timely interventions [67,68]. Given the higher cardiovascular disease risk in patients with diabetes, this predictive capability has important implications for PD patients with coexisting diabetes. Support vector machines, random forest, and deep neural networks have been used for predicting diabetes outcomes [68]. In addition, convolutional neural networks have been effective in identifying diabetic retinopathy from medical images [69], and natural language processing has been employed to identify medication nonadherence and care gaps in diabetes [70].

### AI in peritoneal dialysis management

Fluid management is another area where AI is proving to be useful. Predicting and maintaining the optimal fluid balance is critical for dialysis patients, especially those with diabetes, as fluid overload can exacerbate cardiovascular complications. AI algorithms have been developed to help clinicians optimize ultrafiltration rates in HD and PD patients. By analyzing individual patient data, these models can anticipate fluid overload, thus preventing complications like heart failure [69,71]. In PD patients with diabetes, personalized fluid management is particularly important due to the dual challenges of glycemic control, use of higher concentration dextrose solutions, and fluid retention. AI-driven tools are also being used to customize dialysis prescriptions based on individual patient characteristics. For patients undergoing PD, AI can analyze factors, such as dialysis adequacy, residual kidney function, and diabetes management needs, to tailor treatment regimens. This personalization helps improve overall treatment outcomes, including glycemic control and dialysis efficiency [69]. Moreover, mobile applications and wearable devices equipped with AI have been used to monitor dialysis patients remotely. For PD patients, especially those managing diabetes, real-time tracking of vital signs and glucose levels can alert clinicians to early signs of complications like peritonitis or hypoglycemia. AI tools can tailor dialysis treatment plans by analyzing patient-specific data, ensuring optimal fluid balance, solute clearance, and glucose management, which are critical for improving outcomes in diabetic PD patients. Such remote monitoring tools have been instrumental in ensuring adherence to treatment protocols and facilitating timely interventions [72].

Finally, AI-driven data management platforms integrate electronic health records with predictive models to streamline the decision-making process for dual management of diabetes and dialysis. These systems help clinicians make informed, data-driven decisions in real-time, optimizing care and preventing complications [70,71]. AI applications in diabetes management for PD patients address key concerns such as complication prediction, fluid management, personalized treatment, and real-time monitoring. These advancements, although promising, must overcome challenges like the integration of AI into healthcare systems and ensuring the interpretability of AI models for practical clinical use.

### AI in glucose monitoring and diabetes management

Leveraging advanced technologies, such as CGMs in combination with AI/ML, could revolutionize personalized care for this patient population. These technologies may enable an integrative approach that combines real-time data analytics with precision medicine to tailor treatment protocols that meet the unique needs of each patient (Figure 1). The application of fourth-generation CGMs for real-time tracking of glucose levels in patients with diabetes undergoing PD has been shown to provide detailed measurements of glucose variability, mean sensor glucose, and time within glucose ranges [38]. Access to these additional data analytics may allow for better management of glycemic control. CGM offers significant advantages over traditional HbA1c testing because HbA1c cannot indicate daily glucose variability, a common complication experienced by patients with diabetes during PD treatment due to the use of glucose-rich dialysate [36].

Although there has been an increase in the integration of AI and ML in diabetes care [73], the use of AI/ML is still emerging in a clinical context. Based on the training of retrospective datasets, AI/ML models show the potential to predict a wide range of criteria for diagnostic and treatment purposes, including genomic-metabolomic biomarker signatures in patients with diabetes who are predisposed to DKD, suitable dialysis modalities, PD technique failure and mortality, classification of PD effluent metabolites, and identifying specific immune responses in acute peritonitis [74]. These applications highlight the potential for AI/ML to help estimate risks to better facilitate the management of PD treatment. The next step in the evolution of AI/ML in the care of patients with diabetes receiving PD involves strengthening these findings through clinical validation.

### AI and wearables and artificial implantable kidney

In addition to advanced data analysis, the application of various technologies to maintain patient–clinician communication is needed for enhancing guidance and self-management of PD sessions. This is because patients must perform complex medical procedures independently at home. The use of an AI-driven chatbot has been shown to support this patient population by providing immediate access to information for improved self-care abilities and reduced infection rates [75]. Additionally, remote patient management programs allow for automatic transmission and monitoring of certain PD treatment data and are considered essential for flagging interventions and ensuring patient adherence to treatment prescription [76] (Figure 1). Future technologies, such as wearable or implantable PD devices, could also transform traditional CKD treatment [77]. Miniaturized wearable artificial kidneys based on sorbent technology to regenerate dialysate, allowing for continuous flow PD and increased mobility for patients, could soon be approved for clinical use [78].

## Limitations

### Limitations of multiomics approaches

Multiomics approaches offer significant opportunities to enhance nutrition control in PD patients with diabetes by integrating genomic, metabolomic, and microbiome data to tailor dietary recommendations and optimize metabolic outcomes. These approaches could help identify individual responses to dialysis treatments and dietary interventions, allowing for more precise control of protein-energy wasting, glycemic fluctuations, and cardiovascular disease risk. However, the limitations of multiomics in this setting include the high cost, complexity of data interpretation, and the need for more standardized protocols, which hinder their widespread clinical application and integration into routine care for PD patients. PD presents significant challenges in managing the nutritional needs of patients, particularly those with diabetes, due to protein loss, glucose metabolism disturbances, and fluid imbalances, all of which require precise and individualized dietary modifications. PD patients, especially those with diabetes, often experience Protein Energy Wasting due to increased protein losses in the dialysate, which can lead to malnutrition if not properly addressed. This necessitates a higher protein intake although simultaneously balancing electrolyte disturbances such as hyperkalemia, hyperphosphatemia, and sodium imbalances. In PD patients with diabetes, the glucose content in dialysis solutions poses an additional challenge, as it can lead to significant hyperglycemia, further complicating glycemic control. Managing this requires not only careful insulin adjustment but also monitoring the long-term risk of hyperinsulinemia, which can exacerbate cardiovascular complications and contribute to weight gain. Moreover, these patients must adhere to strict fluid restrictions to prevent volume overload, which can exacerbate hypertension and increase the risk of heart failure. The variability in daily glucose absorption from the PD solution also complicates dietary planning and insulin dosing, leading to unpredictable fluctuations in blood glucose levels. CGMs are particularly critical in this population, but their consistent use is often limited by the costs, access to technology, and practical challenges in routine care. As a result, managing glucose fluctuations induced by the glucose-rich dialysate remains a major hurdle in achieving optimal glycemic control for PD patients with diabetes, underscoring the need for tailored dietary and medical interventions.

The clinical application of genetic polymorphisms presents valuable opportunities for personalized medicine by enabling tailored interventions based on an individual's genetic profile [79], particularly in predicting disease risk and optimizing therapeutic strategies. However, the effectiveness of these applications is constrained by the complexity of gene–environment interactions, where lifestyle factors, such as diet, physical activity, and environmental exposures, modulate genetic expression, making it difficult to predict health outcomes solely from genetic variants. Additionally, the lack of comprehensive data across diverse populations limits the generalizability of findings, often leading to incomplete or biased insights, and raises concerns about equitable implementation of personalized medicine across different demographic groups. This gap can hinder the implementation of genetic testing in personalized medicine, where individualized treatment plans rely on understanding gene–environment interactions.

The microbiome presents a promising opportunity in the management of diabetes in PD, as it could offer novel insights into personalized dietary interventions and metabolic regulation, potentially improving glycemic control and reducing inflammation. Modulating the gut microbiome through probiotics or dietary changes could help mitigate complications such as cardiovascular disease and protein-energy wasting in this population. However, the limitations of microbiome research in PD patients include significant variability in microbiome composition due to factors like dialysis method, medication use, and dietary habits, making it difficult to establish consistent therapeutic approaches. Additionally, the lack of standardized protocols and the dynamic nature of the microbiome pose challenges for integrating microbiome-based strategies into routine clinical practice. Incorporation of gut microbiome data into clinical practice is similarly limited by inconsistent methodologies and a lack of standardized protocols. These issues make it difficult to reliably interpret microbiome data across different patient populations. For example, variations in sample collection techniques, storage conditions, and sequencing technologies can lead to inconsistent results. The inter- and intraindividual variabilities in the microbiome further complicate establishing causality in disease processes, limiting its immediate use in precision diagnostics or treatments. Research into microbiome-targeted therapies, such as probiotics or fecal microbiota transplantation, is ongoing, but standardization is needed before these can be widely adopted.

The application of metabolomics and biomarkers in clinical settings faces similar challenges, particularly due to the multifactorial variability in metabolite levels. These levels can be influenced by external factors, such as diet, physical activity, environmental exposures, and circadian rhythms, making it difficult to achieve reproducible and interpretable results. For instance, metabolites like glucose and insulin fluctuate throughout the day, whereas others, such as SCFAs produced by gut microbiota, can vary based on dietary fiber intake. Additionally, the integration of metabolomics into routine clinical care is hindered by high costs, the need for specialized technical expertise, and the absence of standardized reference ranges for many metabolites. This lack of standardization makes it difficult to compare results across studies or apply findings directly to patient care.

### Limitations of artificial intelligence

One major challenge is data privacy and security. AI applications depend on sensitive health information, such as electronic health records and real-time data from wearable devices, raising concerns about potential privacy breaches.

Another critical limitation is the bias and generalizability of AI models. AI systems are trained on specific datasets, and if these datasets are limited or nonrepresentative, the models may fail to generalize to diverse populations, which is particularly problematic for PD patients with comorbid conditions like diabetes that require specialized data from comparable cohorts. The interpretability of many AI models, especially deep learning algorithms, remains a challenge. These models often operate as “black boxes” making it difficult for healthcare providers to understand the rationale behind their predictions, which can limit their practical use in clinical decision-making for complex conditions. Moreover, integrating AI tools into existing healthcare systems poses logistical difficulties. Many healthcare infrastructures are not designed to accommodate AI-driven tools, and the cost of implementation, along with the need for training healthcare professionals, can be prohibitive.

The quality of the data used in AI models is another significant factor. AI models rely on high-quality, structured data, but in the case of PD patients, incomplete or inconsistent data from electronic medical records or wearable devices can reduce the accuracy and effectiveness of AI predictions. Finally, ethical and regulatory challenges persist, particularly regarding biases in AI-driven decisions, impacts on patient autonomy, and the lack of comprehensive regulatory guidelines governing AI use in chronic disease management, including PD and diabetes.

In conclusion, we propose that the management of diabetes in patients receiving PD could benefit from a comprehensive, integrated approach that encompasses advanced monitoring technologies, genetic insights, biomarkers, and precision nutritional management. The leveraging of multiomics data spanning genomics, proteomics, metabolomics, and microbiome profiles can enable a deeper understanding of individual variation in disease progression and treatment response. This knowledge, combined with clinical validation of modern technological advancements, could support the development of highly tailored treatment plans that address the specific metabolic, genetic, and nutritional needs of each patient. Although not without significant limitations, such an integrative approach can ultimately lead to more effective, personalized treatments, significantly improving the quality of life and clinical outcomes for these patients.

## Author contributions

The authors’ responsibilities were as follows – SM: designed research and outlined the manuscript idea; SM, NMA, TS, PYT: conducted research; SM: wrote the first draft; SM, NMA, TS, PYT: revised subsequent drafts. PYT: provided philanthropic funding for summer students and research associates’ time via the Kidney Life Sciences Institute; SM: had primary responsibility for final content; and all authors: read and approved the final manuscript.

## Funding

The authors reported no funding received for this study.

## Conflict of interest

SM has received funding for advisory board activities, consulting roles, educational grants, travel grants, and speaker/moderator honoraria from the American Academy of Nutrition, World Congress of Aesthetics and Anti-Aging Medicine, Canadian Board of Aesthetic Medicine, Canadian Association of Medical Aesthetics, Canadian Association of Aesthetic Medicine, Los Angeles Multi-Specialty Cosmetic Academy, Allergan, Galderma, Sano, Shire, Genzyme, Gambro, Nutrigenomix, and Abbott. SM has received fellowship, educational, and research funding from Harvard University, the University of Toronto, and Mitacs. SM has provided inkind educational speaker services to the University of Miami Dermatology Department and has served on the Editorial Board of the *Canadian Journal of Kidney Disease and Health*, the ofcial journal of the Canadian Society of Nephrology. SM is an associate editor of the *Canadian Journal of Kidney Disease and Health* and an assistant editor of *BMJ Nutrition, Prevention & Health*.

PT has received funding for advisory board activities and speaker honoraria from Otsuka, Bayer, GSK, Amgen, Boehringer-Ingelheim, Merck, Janssen, Baxter, Fresenius, and Amgen. PT received study grant funding from Janssen for conducting a clinical trial. PT is a co-owner of patents related to the treatment of peritoneal dialysis patients and a co-director of the Kidney Health Life Sciences Institute.

TS has received funding for advisory board activities and speaker honoraria from Sano, Shire, Seaford, Scarborough Health Network, Ontario Renal Network, and the Kidney Health Life Sciences Institute.

The funders and organizations listed above have had no involvement in the writing, interpretation, or conclusions of this manuscript.
